# Editorial: Insights in Microbe and Virus Interactions With Plants: 2021

**DOI:** 10.3389/fmicb.2022.947163

**Published:** 2022-08-02

**Authors:** Marco Scortichini, Elvira Fiallo-Olivé

**Affiliations:** ^1^Council for Agricultural and Economics Research (CREA)-Research Centre for Olive, Fruit and Citrus Crops, Rome, Italy; ^2^Instituto de Hortofruticultura Subtropical y Mediterránea “La Mayora” (IHSM-UMA-CSIC), Consejo Superior de Investigaciones Científicas Algarrobo-Costa, Málaga, Spain

**Keywords:** plant-microbe interaction, plant colonization, detection, climate change, disease control

Both aerial and underground plant organs and tissues are colonized by a composite microbiota that continuously interacts with the host and the surrounding environment. Phytopathogens, beneficial, symbionts, and commensal microbes and viruses are all members of a network that communicate either with the plant or between different microbial taxa through refined molecular mechanisms (Venturi and Bez, 2021). These interactions shape microbial communities according to plant organ/tissue, climatic parameters, and edaphic features (Chaudry et al., 2021). Currently, these interactions should be also assessed considering the climate change scenario (Chaloner et al., 2021). The knowledge of the basic interactions that rule out each specific pathosystem is a fundamental prerequisite to achieving a sustainable control of plant diseases (Leung et al., 2020). Different and complementary techniques can assist such a purpose. Apart from the “omics”, computational software can provide genome-scale metabolic models related to the interplays occurring between co-occurring microbes and viruses, and plants (Lam et al., 2020), whereas detection techniques can benefit from targeted genome sequencing (Maina et al., 2021).

The objective of this Research Topic was to publish high-quality research papers and review articles focusing on the following aspects: (a) new insights on plant-microbes/virus molecular interaction; (b) plant colonization; (c) detection; (d) sustainable control.

This Research Topic contains 12 research papers that cover different aspects of viral, bacterial, and fungal pathogens in relation to plant-microbe molecular interaction, plant colonization, detection, or disease control. In addition, it contains a meta-analysis review that concerns the microbe-mediated thermotolerance in plants.

## Plant-Microbe/Virus Molecular Interaction

The study by Zhang et al. analyzed the function of nuclear export signals (NESs) and nuclear localization signals (NLSs) of the NIb protein of turnip mosaic virus. NES and NLS are key signatures of proteins for controlling nuclear import and export. Two functional NESs and one functional NLS were identified in the NIb protein. Mutation of the identified functional NESs or NLS inhibited viral RNA accumulation and systemic infection. Exportin 1 (XPO1), a nuclear export receptor that binds directly to cargo proteins harboring a leucine-rich NES and translocates them to the cytoplasm, was found to contain two NIb-binding domains, which recognize the NLS and NES of NIb, respectively, to mediate the nucleocytoplasmic transport of NIb and promote viral infection.

The expression of miRNAs in *Nicotiana benthamiana* in response to the infection of tobacco curly shoot virus (TbCSV) *via* small RNAs sequencing was studied by Wu et al. The results showed that 15 up-regulated miRNAs and 12 down-regulated miRNAs were differentially expressed, and nbe-miR167b-3p was down-regulated. The overexpression of nbe-miR167b-3p attenuated leaf curling symptoms of TbCSV and decreased viral DNA accumulation. However, suppression of nbe-miR167b-3p expression enhanced the symptoms and accumulation of the virus. The silencing of PRCP, the target gene of nbe-miR167b-3p caused weakened viral symptoms and DNA accumulation of TbCSV in the plants. Overall, this study clarified the effect of nbe-miR167b-3p on plant defense during TbCSV infection.

A new spectroscopic fluorescence non-destructive method for the *in vivo* detection of the early events in the interaction between compatible and incompatible bacterial pathogens and host plants was provided by Agati et al. It is based on the elaboration of the fluorescence excitation spectra recorded at different times during the elicitation process. The phytoalexin scopoletin F _385−460_ fluorescence has been proven as a marker for plant-bacteria interaction. The outcome of the present study can be exploited to define a screening technique for the early diagnosis of plant diseases, even in the field by using suitable portable fluorescence sensors.

The compatible and incompatible interaction between *Phytopthora infestans* and potato was studied by Zhu et al. through a metabolomic approach and a resistant and a susceptible cultivar. A total of 819 metabolites were identified and quantified. Resistant and sensitive potato cultivars had different metabolomic responses toward *P. infestans*, and the metabolic differences were mainly observed after 48 hpi. The resistant potato cultivar had higher levels of salicylic acid and several upstream phenylpropanoid biosynthesis metabolites, triterpenoids, and hydroxycinnamic acids and their derivatives, such as sakuranetin, ferulic acid, ganoderic acid Mi, lucidenic acid D2, and caffeoylmalic acid.

Transcriptomic analyses and resistance and susceptible non-maize cultivars were utilized to assess the pathogenicity behavior of *Bipolaris maydis* that causes “maize leaf blight” (Meshram et al.). The majority of highly differentially expressed genes were associated with mitochondria, cell wall and chitin synthesis, sugar metabolism, peroxidase activity, mitogen-activated protein kinase activity, and shikimate dehydrogenase. The biosynthetic pathways for secondary metabolism, antibiotics, and carbon metabolism of fungus were highly enriched in the susceptible cultivar during infection. Cell wall synthesis genes related to the synthesis of polyketides, toxins, and putative candidate effector genes were found to be the key compounds underlying the pathogenesis of the *B. maydis* race “O” pathogen.

## Plant Colonization

The bacterial community inhabiting the fruiting body of the saprotrophic and necrotrophic Basidiomycetes *Heterobasidium* species complex, and the associated wood, have been analyzed through the functional annotation of the 16S sequence data (Ren et al.). The study showed that bacteria communities in both substrates experienced a process of a new community reconstruction through the various decay stages. *Sphingomonas* spp. was significantly higher in the fruiting body, and phylum Firmicutes was more dominant in wood tissue. The bacteria community was highly dynamic, and the microbiota activeness, community stability, and functions changed with the decay process. Bacteria appear to spread from the wood tissue of the standing living tree to the fruiting body, but after the tree is killed, bacteria moved from the fruiting body to the wood.

Della Rocca et al. have studied, by means of transmission electron microscope, light and fluorescence microscope combined with histochemical analyses staining, in a time-course approach the induction and the ultrastructure of terpenes and polyphenol synthesis in *Cupressus sempervirens* twigs upon *Seiridium cardinale* infection. The study highlighted the involvement of plastids and endoplasmic reticulum in the production of terpenoids and the consequent secretion of terpenoids directly through the plasma membrane, without exhibiting vesicle formation. Plastids are also involved in the polyphenol production that accumulates in the vacuole.

## Detection

A comparison of the most established high-throughput sequencing (HTS) platform (MiSeq benchtop sequencer-Illumina) with the MinION sequencer (Oxford Nanopore Technologies) for the detection of plant viruses and viroids was carried out (Pecman et al.). Method comparisons were performed on five selected samples, containing two viroids, which were sequenced using nanopore technology for the first time, and 11 plant viruses with different genome organizations. The results obtained in this work showed that when an appropriate sample and library preparation are selected, nanopore MinION sequencing could be used for the detection of plant viruses and viroids with similar performance as Illumina sequencing.

## Disease Control

The work carried out by Taglienti et al. evaluates the antiphytoviral effectiveness of treatments with three essential oils and corresponding hydrosols extracted from *Origanum vulgare, Thymus vulgaris*, and *Rosmarinus officinalis* on *Cucurbita pepo* plants infected by zucchini yellow mosaic virus or tomato leaf curl New Delhi virus (ToLCNDV). Treatments were applied either concurrently or after virus inoculation to ascertain an inhibition or curative activity, respectively. Treatments were effective against ToLCNDV whether applied simultaneously with the inoculation or after and the major inhibition was observed with *O. vulgare* essential oil and hydrosol. The curative activity gave maximum results with all three essential oils and *T. vulgaris* and *R. officinalis* hydrosols.

The effects of biochar (a rich carbon product obtained by pyrolysis of biomass under a limited supply of oxygen) obtained from olive pruning, have been evaluated on tomato seedling growth and response to systemic agents' infection alone or added with the beneficial microorganisms *Bacillus* spp. and *Trichoderma* spp. (Luigi et al.). Biochar seems to promote the development of tomato seedlings without showing any antimicrobial effects on the beneficial soil bacteria at the tomato rhizosphere level and even improving their growth. Biochar at 10–15% and when added with *Trichoderma* spp. caused a reduction of the replication and symptoms of potato spindle tuber viroid. Also, results obtained showed that biochar could contribute to reducing both infection rate and virus replication of tomato spotted wilt virus.

A study regarded the isolation and characterization of bacteriophages obtained from melon and watermelon infected by the destructive bacterium *Acidovorax citrulli*, and the further genomic characterization and evaluation of the systemic persistence and translocation of a phage from the roots to the upper parts of watermelon plants (Gasic et al.). The persistence of the phages in the plant environment upon their release is one of the critical points for their successful utilization in plant disease control, being that UV light is one of the most adverse factors. The study ascertained a persistence and survival of 10 days within the plant post distribution of the phage in the soil, allowing a relevant decrease in the severity of the disease and the survival of the plants. These findings corroborate previous studies on the possibility to control endophytic bacterial pathogens through phages distributed to the root system.

*Fusarium oxysporum* is one of the most dangerous soilborne fungal pathogens, capable of infecting many crops worldwide. Iida et al. assessed the biocontrol activity of non-pathogenic mutants of *F. oxysporum* ff. spp. *melonis* and *lycopersici*, which infect melon and tomato, respectively. The mutants were obtained by disruption of the *FOW*_2_ gene. The pre-inoculation of melon and tomato roots with the mutants allowed to reduce the disease incidence. The study indicated that the ability to reduce the pathogen activity was due to the relevant capability showed by the non-pathogenic mutants to colonize the root system and compete for nutrients with the pathogens.

## Climate Change and Plant Symbionts

A meta-analysis review performed by Dastogeer et al. investigated the interaction of microbial symbionts and plants within a climate change scenario. One of their main conclusions is the lack of experiments that demonstrate how microbes influence plant responses to high temperature stress in the natural environment. Moreover, the Authors indicated that microbial colonization influenced plant growth and physiology, but their effects were more noticeable when the host plants were exposed to high-temperature stress than when they grew under ambient temperature conditions. Another relevant conclusion of the meta-analysis is that, in addition to microbe-plant interaction, microbe-microbe and microbe-environment interaction should be taken into account to understand the stable functions of a particular microbe in its potential application in agricultural settings.

## Author Contributions

All authors listed have made a substantial, direct, and intellectual contribution to the work and approved it for publication.

## Conflict of Interest

The authors declare that the research was conducted in the absence of any commercial or financial relationships that could be construed as a potential conflict of interest.

## Publisher's Note

All claims expressed in this article are solely those of the authors and do not necessarily represent those of their affiliated organizations, or those of the publisher, the editors and the reviewers. Any product that may be evaluated in this article, or claim that may be made by its manufacturer, is not guaranteed or endorsed by the publisher.
